# Flightless Females in the Neotropical Moth Genus *Cataspilates* Warren (Lepidoptera: Geometridae) †

**DOI:** 10.3390/insects13111003

**Published:** 2022-10-31

**Authors:** Héctor A. Vargas

**Affiliations:** Departamento de Recursos Ambientales, Facultad de Ciencias Agronómicas, Universidad de Tarapacá, Arica 1000000, Chile; lepvargas@gmail.com or havargas@academicos.uta.cl

**Keywords:** Andes, apterous, brachypterous, DNA barcodes, flightless, sexual dimorphism

## Abstract

**Simple Summary:**

Adults are winged and able to fly in most Lepidoptera species. However, adults of some species are unable to fly, since their wings are reduced or absent. In the moth family Geometridae (geometrid moths), wing reduction is restricted to females. The aim of this study is to provide the first record of flightless females for a South American genus of the tribe Boarmiini of the geometrid moth family through the description of a new species from the arid Andes of northern Chile. Analysis of DNA sequences was used to confirm that males and females examined in this study are conspecific. This contribution provides a new opportunity to improve the understanding of the evolution of wing reduction in geometrid moths.

**Abstract:**

Although adults are winged and able to fly in most Lepidoptera species, they are apterous or brachypterous and unable to fly in others, such as the flightless females of some geometrid moths. Records of flightless females in the highly diverse and widespread tribe Boarmiini (Geometridae: Ennominae) are mainly restricted to some Nearctic and Palearctic genera. The aim of this study is to provide the first record of flightless females for *Cataspilates* Warren, 1897, a Boarmiini genus endemic to the Neotropical Region, through the description of *Cataspilates marceloi* sp. nov. from the arid highlands of the western slopes of the Andes of northern Chile. DNA barcodes confirmed the conspecificity of brachypterous females and winged males reared from larvae collected on the native shrub *Adesmia spinosissima* (Fabaceae). This contribution represents the first female description for *Cataspilates* and provides a new opportunity to improve the understanding of the evolution of flightlessness in geometrid moths.

## 1. Introduction

Although most species of Lepidoptera have winged adults with flight ability, species with apterous or brachypterous adults unable to fly occur in some families [1,2]. Flightlessness in the moth family Geometridae is restricted to females and occurs in distantly related lineages belonging to different subfamilies, suggesting multiple evolutionary origins [2,3]. Besides wing reduction, flightless geometrid females may have other morphological peculiarities such as reduced mouthparts and tympanal organs [4], or highly modified thoracic exoskeleton and muscles [5]. The transition to flightless females in lineages of geometrid moths could have been favored after the colonization of stable forest habitats followed by the evolution of key traits that reduce the importance of oviposition site selection and adult feeding [6]. Flightless females originated at least four times independently in the highly diverse and widespread geometrid moth tribe Boarmiini (Ennominae), with the oldest event dated to about 37 Mya [7]. The frequency of wing reduction evolution in Boarmiini is higher than in any other tribe of Geometridae, suggesting that its members are predisposed for flightlessness to evolve [8]. However, records of flightless females in Boarmiini are mainly restricted to Nearctic and Palearctic genera [7,8]; no cases have been recorded for the Neotropical fauna of this tribe.

*Cataspilates* Warren, 1897 is a small Neotropical geometrid moth genus recognized as a member of Boarmiini by Pitkin [9], but this was recorded without detailed justification. Based on the species type *Cataspilates grisescens* Warren, 1897, described from Bogotá, Colombia, she [9] indicated that the genus is distinguished by the male genitalia morphology and suggested that the broad, bilobed juxta represents a putative apomorphy. The only specimen of *Cataspilates* recorded in Chile, a male adult from the western slopes of central Andes, was identified as *C. grisescens*, but the need for further studies to clarify its taxonomic status was highlighted, since its genitalia showed subtle morphological differences from those of the type material [10]. Additional males of *Cataspilates* from Chile were recently reared from larvae collected on a native shrub in the same geographic area as the previous record. Examination of their genitalia revealed identical morphology to that of the only specimen previously known from this country, suggesting that all these represent an undescribed species morphologically different from *C. grisescens*. Surprisingly, brachypterous females were reared along with the winged males. As females of *Cataspilates* were unknown before this discovery [9], the conspecificity of the brachypterous females and winged males was assessed using mitochondrial DNA sequences.

The aim of this study is to provide the first record of female flightlessness for *Cataspilates* through the description of a new species from the arid highlands of central Andes and to assess its phylogenetic relationships using DNA sequence analysis.

## 2. Materials and Methods

The moths examined in this study were reared from larvae collected on the native shrub *Adesmia spinosissima* (Fabaceae) near Murmuntani (18°20′43″ S, 69°33′06″ W) and Socoroma (18°16′42″ S, 69°34′15″ W) villages, Parinacota Province, at about 3400–3500 m elevation on the western slopes of the Andes of northern Chile. The abdomen of each adult was removed and placed in hot KOH 10% for a few minutes for genitalia dissection. Genitalia were stained with Eosin Y and Chlorazol Black and mounted on slides with Euparal. Photos of the male and female were taken with a Sony CyberShot DSC-HX200V digital camera and a Leica Flexacam C1 digital camera attached to a Leica M125 stereomicroscope, respectively. Photos of the genitalia were taken with a Leica MC170 HD digital camera attached to a Leica DM1000 LED light microscope. Each image was constructed with about 5–10 photos assembled with the software Helicon Focus 8. The specimens studied will be deposited in the “Museo Nacional de Historia Natural de Santiago” (MNNC), Santiago, Chile, and the “Colección Entomológica de la Universidad de Tarapacá” (IDEA), Arica, Chile.

Genomic DNA was extracted from legs of one male and one female using the QIAamp^®^ Fast DNA Tissue Kit, following the manufacturer’s instructions, and sent to Macrogen Inc. (Seoul, South Korea) for purification, PCR amplification and sequencing of the 658 bp length barcode region [11] using the primers LCO1490 and HCO2198 [12]. The PCR program was of 5 min at 94 °C, 35 cycles of 30 s at 94 °C, 30 s at 47 °C, 1 min at 72 °C and a final elongation step of 10 min at 72 °C.

Phylogenetic relationships of *Cataspilates* have still not been explored, but male genitalia morphology suggests some closeness to *Glenoides* McDunnough, 1922 [13], *Glena* Hulst, 1896 [14] and *Physocleora* Warren, 1897 [9]. Representatives of these three genera grouped with members of *Anavitrinella* McDunnough, 1922 and *Odysia* Guenée, (1858) in a recent molecular phylogenetic study of Boarmiini [7]. Accordingly, sequences of these genera (type species if possible) were downloaded from the BOLD database [15] to assess the phylogenetic relationships of the new species through a maximum likelihood (ML) analysis (Table 1). As *Tornos* Morrison, 1875, was sister to this group in the analysis of [7], sequences of this genus were also included in the alignment, as well as the nearest neighbor sequence of the new *Cataspilates* in BOLD, “*Physocleroa*” sp. (GEOCO032-20) from Colombia. The software MEGA11 [16] was used to perform sequence alignment through the ClustalW method and to estimate genetic distances with the Kimura 2-Parameter (K2P) method. Substitution saturation of the alignment was assessed with the Xia test [17] using the software DAMBE7 [18]. The ML analysis was performed with the software IQTREE 1.6.12 [19] in the web interface W-IQ-TREE [20]. Data were partitioned to codon position and TN+F+I, F81+F and HKY+F+G4 were selected as the best-fit models for 1st, 2nd and 3rd partitions, respectively, in ModelFinder [21]. Branch support was calculated with 1000 replicates of ultrafast bootstrap, UFBoot2 [22]. The unrooted tree obtained was visualized in FigTree [23] to root on *Tornos*.

## 3. Results

### 3.1. Molecuar Analysis

**Figure 1:**
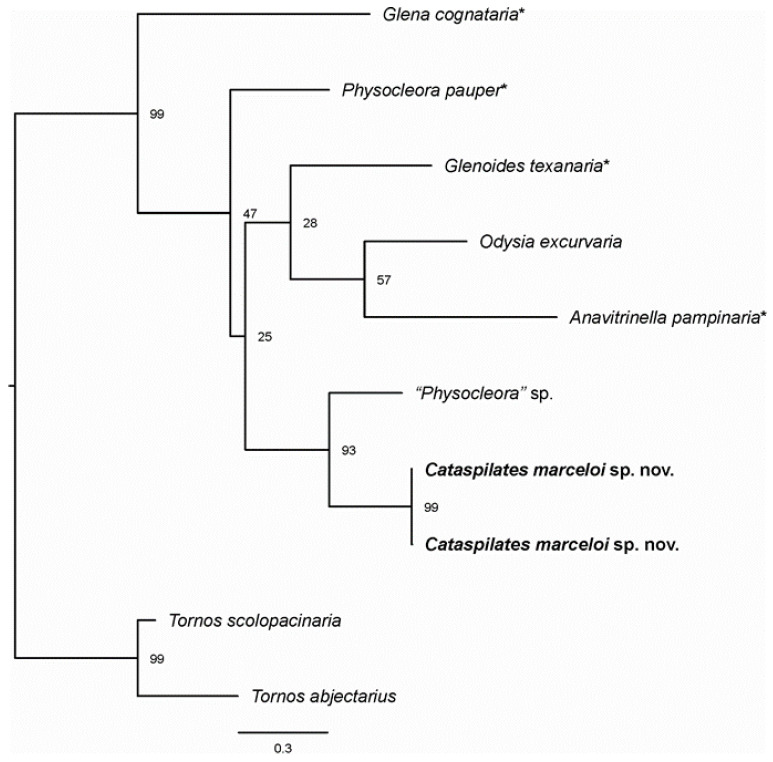
Maximum likelihood tree of *Cataspilates marceloi* sp. nov. (bold) and close Neotropical genera of Boarmiini based on mitochondrial DNA sequences. The asterisk indicates type species of the respective genus. Numbers indicate UFBoot2 support values (1000 replicates).

DNA barcodes of 658 bp length were obtained from one male (BOLD: CATMA001-22) and one female (BOLD: CATMA002-22) of the new species. The distance between them was 0.2% (K2P). The two sequences were aligned with eight others from BOLD and trimmed to 600 bp due to the shorter sequence of “*Physocleora*” sp. The alignment was suitable for phylogenetic analysis, as no evidence of stop codons was detected and the index of substitution saturation was smaller than the critical value (ISS < ISS.C; *p* < 0.001) in the Xia test. Genetic distance of the new species to its nearest neighbor “*Physocleora*” sp. was 5.9–6.1%. The support values for the monophyly of the new species and for its clustering with “*Physocleora*” sp. in the ML analysis were 99 and 93% UFBoot2, respectively (Figure 1).

### 3.2. Taxonomy

*Cataspilates marceloi* sp. nov.

Zoobank link: urn:lsid:zoobank.org:act:421C1313-DF0A-4E33-8240-0D76DA32CDB5.

**Figure 2:**
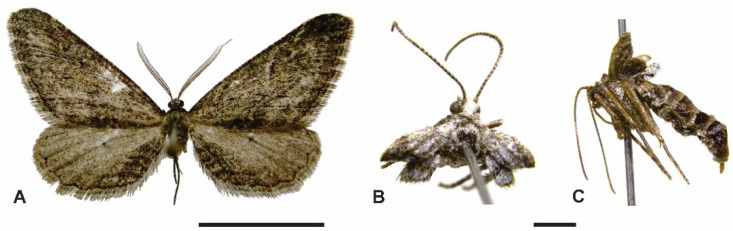
Habitus of *Cataspilates marceloi* sp. nov. (**A**) Holotype male, dorsal. (**B**) Paratype female, dorsal. (**C**) Paratype female, lateral. Scale bars 10, 2 mm, respectively.

**Figure 3:**
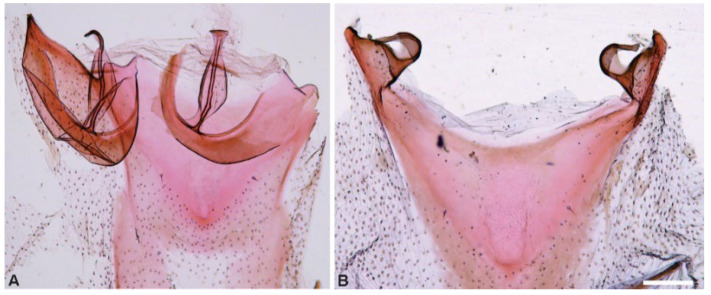
Tympanal organs of *Cataspilates marceloi* sp. nov. (**A**) Male, ventral. (**B**) Female, ventral. Scale bar 0.2 mm.

**Figure 4:**
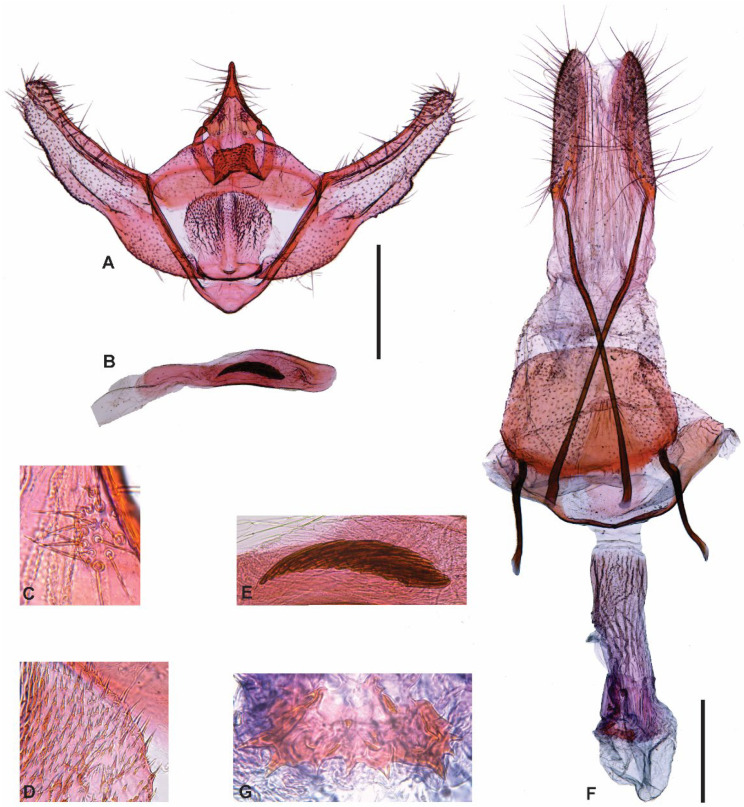
Genitalia of *Cataspilates marceloi* sp. nov. (**A**) Male genitalia, ventral, phallus removed. (**B**) Phallus, lateral. (**C**) Right socius. (**D**) Sculpturing of right lobe of the juxta. (**E**) Cornuti. (**F**) Female genitalia. (**G**) Signum. Scale bars 0.5 mm.

**Figure 5:**
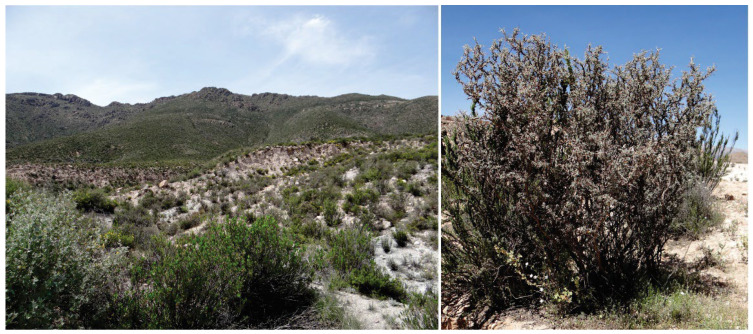
The type locality near Murmuntani village (**left**) and host plant *Adesmia spinosissima* (Fabaceae) (**right**) of *Cataspilates marceloi* sp. nov. in the Andes of northern Chile.

Figure 2, Figure 3, Figure 4 and Figure 5.

*Cataspilates grisescens* Warren, 1897, misidentification [10].

Type material. HOLOTYPE, male, CHILE: Chile, Parinacota, Murmuntani, adult emerged May 2021, ex larva collected April 2021 on *Adesmia spinosissima*, H.A. Vargas leg.; IDEA-LEPI-2022-011; genitalia slide HAV-1529 (MNNC).

Paratypes, Chile. Three females, same data as for holotype, except for M. Vargas-Ortiz leg.; IDEA-LEPI-2022-012 to IDEA-LEPI-2022-014; genitalia slides HAV-1518, HAV-1530, HAV-1531 (IDEA). One male, Chile, Parinacota, Socoroma, adult emerged January 2020, ex larva collected December 2019 on *Adesmia spinosissima*, H.A. Vargas leg.; IDEA-LEPI-2022-015; genitalia slide HAV-1345 (IDEA).

Diagnosis. The male genitalia of *C. marceloi* sp. nov. are remarkably similar to those of the type species *C. grisescens*. However, in *C. marceloi* sp. nov. the tip of the costa lightly exceeds the cucullus, the distal half of the cucullus is almost parallel to the dorsal margin of the costa, and the vesica has a group of small, stout spine-like cornuti. In contrast, in *C. grisescens* the tip of the costa clearly exceeds the cucullus, the distal half of the cucullus is broadly rounded, and the vesica has a broad pad-like cornutus, a curved spine-like cornutus longer than phallus diameter and a group of small, narrow spine-like cornuti. The female of *C. grisescens* remains unknown, impeding comparisons with *C. marceloi* sp. nov.

Male (Figure 2A and Figure 3A). Winged, forewing length 15.3–16.7 mm. Head. Vertex mostly brownish gray with scattered blackish gray scales. Frons blackish gray with brownish gray transverse stripe on ventral margin. Antenna bipectinate, brownish gray dorsally. Labial palpus brownish gray. Haustellum well developed. Thorax. Mostly brownish gray with scattered blackish gray scales. Legs with brownish gray and blackish gray scales intermixed. Forewing mostly with brownish gray and blackish gray scales intermixed and scattered creamy white scales; a narrow, sinuous line slightly differentiated from middle anal margin to apex of discal cell; a small blackish gray discal spot poorly differentiated. Hindwing mostly brownish gray with small blackish gray discal spot and short blackish gray lines near anal margin. Abdomen. Mostly brownish gray with scattered blackish gray and creamy white scales. Tympanal organ normally developed.

Male genitalia (Figure 4A–E). Tegumen semicircular in dorsal view, anterior margin slightly concave, posterior margin broadly convex. Uncus triangular, slightly narrowed at junction with tegumen, apex rod-like with rounded tip. Saccus triangular with broadly rounded anterior tip, posterior margin slightly convex. Valva narrow; costal margin slightly concave, ventral margin sinuous; costa well sclerotized with apex slightly dilated and a few short, straight setae; sacculus slightly sclerotized, ventral margin broadly convex; cucullus mostly membranous, almost reaching apex of costa, proximal half of ventral margin broadly convex, distal half almost parallel to dorsal margin of costa. Juxta bilobed, anterior margin straight, lateral margins broadly rounded, posterior half with microtrichiae. Phallus cylindrical, abruptly narrowing toward coecum; vesica with a group of small, stout spine-like cornuti.

Female (Figure 2B,C and Figure 3B). Brachypterous, forewing length 2.5–2.6 mm. Head. Vertex whitish gray. Frons blackish gray. Antenna filiform, brownish gray. Labial palpus brownish gray. Haustellum well developed. Thorax. Mostly whitish gray with scattered brownish gray scales. Legs with whitish gray and brownish gray scales intermixed. Forewing mostly with whitish gray with scattered brownish gray scales. Hindwing slightly shorter than forewing, frenulum absent, mostly with whitish gray with scattered brownish gray scales. Abdomen. Mostly whitish gray with scattered brownish gray and blackish gray scales. Tympanal organ poorly developed.

Female genitalia (Figure 4F,G). Papillae anales membranous, narrow, elongated, with setae. Apophyses posteriores rod-shaped, about twice the length of papillae anales. Apophyses anteriores rod-shaped, about one third the length of apophyses posteriores, with strong curvature near base. Lamella antevaginalis a narrow, slightly curved transverse rod, with flat expanded tips. Lamella postvaginalis a flat longitudinal stripe similar in length to apophyses anteriores, anterior half parallel-sided, posterior half with posteriorly convergent margins. Antrum membranous, about half the length of lamella postvaginalis. Ductus bursae about 1.5 times the length of apophyses anteriores, slightly sclerotized, longitudinally striated. Corpus bursae spherical, membranous, slightly wider than ductus bursae, with ellipsoidal signum with dentate anterior margin. Ductus seminalis arising from the middle of ductus bursae.

Etymology. The name of the species is dedicated to my dear brother Marcelo Felipe Vargas Ortiz, for his passion for nature and for his kind company in the fieldwork on many occasions. As a part of our collaborations, he reared the three brachypterous females used in this study.

Geographic distribution. Distribution records of *C. marceloi* sp. nov. are restricted to western slopes of the Andes of northern Chile between about 3400–3500 m elevation.

Host plant. The shrub *Adesmia spinosissima* (Fabaceae) (Figure 5) is the only host plant known for *C. marceloi* sp. nov.

## 4. Discussion

DNA barcoding is a valuable tool for biodiversity studies [11]; its usefulness was already recognized in studies dealing with Neotropical geometrid moths [24,25,26,27,28]. This tool provides support for accurate taxonomic identification of immature stages [29], and to associate sexes of a given geometrid species [30], among other things. Although females and males of *C. marceloi* sp. nov. were reared from larvae collected on the same host plant and locality, the extreme sexual dimorphism of the adults could generate some doubt about whether they belong to the same species. However, the genetic distance between them is in the intraspecific range generally recorded for geometrid moths [31,32,33], and the monophyly of the cluster containing the two haplotypes was well supported [22], confirming that the brachypterous females and winged males of *Cataspilates* studied here are conspecific.

The females of *C. marceloi* sp. nov. are the first ones recorded for the genus, whose previous knowledge was exclusively based on males [9]. The inability of the brachypterous females to fly might have hindered their earlier discovery, because early surveys for geometrid moths in the Neotropics were performed mainly using light traps, which primarily attract flying insects. Searches for larvae in some recent studies have enabled a better understanding of additional aspects of the natural history of Neotropical geometrids [34,35,36,37,38]. The brachypterous females of *C. marceloi* sp. nov. were discovered only when larvae were collected and reared to adult stage, almost 50 years after the first winged male was collected at light [10]. Surveys for larvae on native plants at the type localities of other species of *Cataspilates* would be extremely helpful to verify if they also have flightless females.

Due to the limited taxon sampling and UFBoot2 value slightly lower than the recommended for well-supported clades [22], clustering of *C. marceloi* sp. nov. with “*Physocleora*” sp. should be viewed with caution. It was surprising that “*Physocleora*” sp. did not cluster with the type species of this Neotropical genus in the ML analysis, because phylogenetic analyses based on mitochondrial DNA sequences generally provide adequate generic assignment for geometrid moths [32,39]. However, recent molecular phylogenetic studies suggest potential polyphyly of *Physocleora* [7,40], making the result understandable. Further studies are needed to assess the generic assignment of “*Physocleora*” sp., starting with the examination of the morphology of its genitalia. Furthermore, although *C. marceloi* sp. nov. appears well placed in *Cataspilates* based on the morphological definition [9], analysis of sequences of the type species and additional members of this genus would provide valuable insights regarding their phylogenetic relationships.

Multiple independent evolutionary events toward flightless females have been recognized in the family Geometridae [3,6,8], at least four of which occurred in lineages of Boarmiini [7]. Although further studies are needed to improve the understanding of the phylogenetic relationships of *Cataspilates*, the currently available information suggests that female flightlessness in this genus would have arisen as an event independent of the four already recognized in the tribe. Along with phylogenetic analyses based on a wider taxon sampling and additional markers, studies dealing with the morphology and natural history of *Cataspilates* and allied genera are encouraged, because detailed knowledge of these aspects would be extremely helpful to understand the evolution of female flightlessness in this Neotropical genus of geometrid moths.

## Figures and Tables

**Table 1:** DNA barcode sequences used in the molecular analysis. The asterisk indicates type species of the respective genus. Sequences generated in this study in bold.

Species	BOLD Accession
*Anavitrinella pampinaria* (Guenée, 1858) *	DUNLP059-08
***Cataspilates marceloi* sp. nov.**	**CATMA001-22**
***Cataspilates marceloi* sp. nov.**	**CATMA002-22**
*Glena cognataria* (Hübner, 1831) *	LNAUS3126-13
*Glenoides texanaria* (Hulst, 1888) *	BBLSW899-09
*Odysia excurvaria* (Warren, 1907)	NGEOB458-10
*Physocleora pauper* Warren, 1897 *	BCIGE031-10
*“Physocleora”* sp.	GEOCO032-20
*Tornos abjectarius* (Hulst, 1887)	TXLEP1124-20
*Tornos scolopacinaria* (Guenée, 1858)	GWNC094-07

## Data Availability

The specimens studied will be deposited in the “Museo Nacional de Historia Natural de Santiago” (MNNC), Santiago, Chile, and the “Colección Entomológica de la Universidad de Tarapacá” (IDEA), Arica, Chile. The sequences generated in this study are deposited in BOLD under accessions CATMA001-22 and CATMA002-22. This article and the nomenclatural act it contains are registered in ZooBank, the online registration system for the ICZN (International Code of Zoological Nomenclature). The links to ZooBank registrations are indicated in the text of the article.

## References

[1] Heppner J.B. (1991). Brachyptery and aptery in Lepidoptera. Trop. Lepid..

[2] Sattler K. (1991). A review of wing reduction in Lepidoptera. Bull. Br. Mus. Nat. Hist. Entomol..

[3] Yamamoto S., Sota T. (2007). Phylogeny of the Geometridae and the evolution of winter moths inferred from a simultaneous analysis of mitochondrial and nuclear genes. Mol. Phylogenet. Evol..

[4] Petschenka G., Tavakoli M., Trusch R. (2006). Description of the unknown female of *Agriopis beschkovi* Ganev, 1987 (Geometridae: Ennominae), and illustration of the larvae. Nota Lepidopterol..

[5] Liu S.-P., Wipfler B., Niitsu S., Beutel R.G. (2017). The thoracic anatomy of the male and female winter moth *Nyssiodes lefuarius* (Lepidoptera: Geometridae) and evolutionary changes in the thorax of moths and butterflies. Org. Divers. Evol..

[6] Snäll N., Tammaru T., Wahlberg N., Viidalepp J., Ruohomaki K., Savontaus M.L., Huoponen K. (2007). Phylogenetic relationships of the tribe Operophterini (Lepidoptera, Geometridae): A case study of the evolution of female flightlessness. Biol. J. Linn. Soc..

[7] Murillo-Ramos L., Chazot N., Sihvonen P., Õunap E., Jiang N., Han H., Clarke J.T., Davis R.B., Tammaru T., Wahlberg N. (2021). Molecular phylogeny, classification, biogeography and diversification patterns of a diverse group of moths (Geometridae: Boarmiini). Mol. Phylogenet. Evol..

[8] Wahlberg N., Snäll N., Viidalepp J., Ruohomäki K., Tammaru T. (2010). The evolution of female flightlessness among Ennominae of the Holarctic forest zone (Lepidoptera, Geometridae). Mol. Phylogenet. Evol..

[9] Pitkin L.M. (2002). Neotropical Ennominae moths: A review of the genera (Lepidoptera: Geometridae). Zool. J. Linn. Soc..

[10] Vargas H.A., Hausmann A. (2008). Additions to the geometrid fauna (Lepidoptera: Geometridae) of Chile. Neotrop. Entomol..

[11] Hebert P.N., Cywinska A., Ball S., de Waard J. (2003). Biological identifications through DNA barcodes. Proc. R. Soc. Lond. B Biol. Sci..

[12] Folmer O., Black M., Hoeh W., Lutz R., Vrijenhoek R. (1994). DNA primers for amplification of mitochondrial cytochrome c oxidase subunit I from diverse metazoan invertebrates. Mol. Mar. Biol. Biotechnol..

[13] McDunnough J. (1920). Studies in North American Cleorini (Geometridae). Can. Dept. Agric. Bull..

[14] Rindge F.H. (1965). A revision of the Nearctic species of the genus *Glena* (Lepidoptera, Geometridae). Bull. Am. Mus. Nat. Hist..

[15] Ratnasingham S., Hebert P.D.N. (2007). BOLD: The Barcode of Life Data System. Mol. Ecol. Notes.

[16] Tamura K., Stecher G., Kumar S. (2021). MEGA11: Molecular evolutionary genetics analysis version 11. Mol. Biol. Evol..

[17] Xia X., Xie Z., Salemi M., Chen L., Wang Y. (2003). An index of substitution saturation and its application. Mol. Phylogenet. Evol..

[18] Xia X. (2018). DAMBE7: New and improved tools for data analysis in molecular biology and evolution. Mol. Biol. Evol..

[19] Nguyen L., Schmidt H., von Haeseler A., Minh B.Q. (2015). IQ-TREE: A fast and effective stochastic algorithm for estimating maximum-likelihood phylogenies. Mol. Biol. Evol..

[20] Trifinopoulos J., Nguyen L., von Haeseler A., Minh B.Q. (2016). W-IQ-TREE: A fast online phylogenetic tool for maximum likelihood analysis. Nucleic Acids Res..

[21] Kalyaanamoorthy S., Minh B.Q., Wong T.K., Haeseler A., Jermiin L.S. (2017). ModelFinder: Fast model selection for accurate phylogenetic estimates. Nat. Methods.

[22] Hoang D.T., Chernomor O., von Haeseler A., Minh B.Q., Vinh L.S. (2017). UFBoot2: Improving the Ultrafast Bootstrap Approximation. Mol. Biol. Evol..

[23] Rambaut A. (2018). FigTree v1.4.4. https://github.com/rambaut/figtree/releases.

[24] Brehm G., Hebert P.D.N., Colwell R.K., Adams M.O., Bodner F., Friedemann K., Möckel L., Fiedler K. (2016). Turning up the heat on a hotspot: DNA barcodes reveal 80% more species of geometrid moths along an Andean elevational gradient. PLoS ONE.

[25] Hausmann A., Parra L.E. (2009). An unexpected hotspot of moth biodiversity in Chilean northern Patagonia (Lepidoptera, Geometridae). Zootaxa.

[26] Moraes S.S., Montebello Y., Stanton M.A., Yamaguchi L.F., Kato M.J., Freitas A.V.L. (2021). Description of three new species of Geometridae (Lepidoptera) using species delimitation in an integrative taxonomy approach for a cryptic species complex. PeerJ.

[27] Murillo-Ramos L., Sihvonen P., Brehm G., Ríos-Malaver I.C., Wahlberg N. (2021). A database and checklist of geometrid moths (Lepidoptera) from Colombia. Biodivers. Data J..

[28] Strutzenberger P., Brehm G., Fiedler K. (2011). DNA barcoding-based species delimitation increases species count of *Eois* (Geometridae) moths in a well-studied tropical mountain forest by up to 50%. Insect Sci..

[29] Gossner M.M., Hausmann A. (2009). DNA barcoding enables the identification of caterpillars feeding on native and alien oak (Lepidoptera: Geometridae). Mitt. Munch. Entomol. Ges..

[30] Guerrero J.J., Hausmann A., Rubio R.M., Garre M., Ortiz A.S. (2022). First description of the male and DNA barcode of *Euphyia vallantinaria* (Oberthür, 1890) from the Iberian Peninsula (Lepidoptera, Geometridae, Larentiinae). Nota Lepidopterol..

[31] Hausmann A., Haszprunar G., Hebert P.D.N. (2011). DNA barcoding the geometrid fauna of Bavaria (Lepidoptera): Successes, surprises and questions. PLoS ONE.

[32] Vargas H.A., Solis M.A., Vargas-Ortiz M. (2022). The South American moth *Rheumaptera mochica* (Dognin, 1904) (Lepidoptera, Geometridae, Larentiinae) rediscovered after more than a century of anonymity. ZooKeys.

[33] Wanke D., Hausmann A., Rajaei H. (2019). An integrative taxonomic revision of the genus *Triphosa* Stephens, 1829 (Geometridae: Larentiinae) in the Middle East and Central Asia, with description of two new species. Zootaxa.

[34] Bocaz P.A., Parra L.E. (2005). Revisión y bionomía del género *Syncirsodes* Butler 1882 (Lepidoptera: Geometridae). Rev. Chil. Hist. Nat..

[35] Bodner F., Brehm G., Homeier J., Strutzenberger P., Fiedler K. (2010). Caterpillars and host plant records for 59 species of Geometridae (Lepidoptera) from a montane rainforest in southern Ecuador. J. Insect Sci..

[36] Brehm G. (2003). Host-plant records and illustrations of the larvae of 19 geometrid moth species from a montane rain forest in Ecuador (Lepidoptera: Geometridae). Nachr. Entomol. Ver. Apollo.

[37] Marconato G., Dias M.M., Penteado-Dias M.A. (2008). Larvas de Geometridae (Lepidoptera) e seus parasitoides, associadas à Erythroxylum microphyllum St.-Hilaire (Erythroxylaceae). Rev. Bras. Entomol..

[38] Vargas H.A. (2021). On the natural history of *Cosmophyga cortesi* Vargas (Lepidoptera: Geometridae), a little-known geometrid moth of the Atacama Desert. Rev. Bras. Entomol..

[39] Wanke D., Feizpour S., Hausmann A., Viidalepp J., Rajaei H. (2022). Taxonomy and systematics of the enigmatic emerald moth *Xenochlorodes graminaria* (Kollar, 1850) (Lepidoptera: Geometridae), and its assignment to a new genus. Integr. Syst..

[40] Brehm G., Murillo-Ramos L., Sihvonen P., Hausmann A., Schmidt B.C., Õunap E., Moser A., Mörtter R., Bolt D., Bodner F. (2019). New World geometrid moths (Lepidoptera: Geometridae): Molecular phylogeny, biogeography, taxonomic updates and description of 11 new tribes. Arthropod Syst. Phylogeny.

